# Genital cutaneous necrosis: a delayed sequela of intraperitoneal Mitomycin-c

**DOI:** 10.1515/pp-2023-0021

**Published:** 2023-10-13

**Authors:** Sage A. Vincent, Meghan Maceyko, Peter J. Altshuler, Zachary Davis, J. Ryan Mark, Paul H. Chung, Wilbur B. Bowne

**Affiliations:** Department of Surgery, Thomas Jefferson University, Philadelphia, PA, USA; Sidney Kimmel Medical College, Thomas Jefferson University, Philadelphia, PA, USA; Department of Urology, Thomas Jefferson University, Philadelphia, PA, USA

**Keywords:** genital, cutaneous, necrosis, intraperitoneal, Mitomycin C

Genital cutaneous necrosis occurring after abdominopelvic tumor cytoreduction and hyperthermic intraperitoneal chemotherapy (HIPEC) with Mitomycin C (MMC) remains an enigma. Herein, we present a pictorial-based synopsis that draws further attention to the delayed onset, protracted clinical course, highlighting potential mechanisms of injury, pathogenesis and treatment.

A 51-year-old male with a recent diagnosis consistent with low-grade mucinous carcinoma variant-pseudomyxoma peritonei (PMP) underwent cytoreductive surgery (CRS) followed by regional perfusion/HIPEC using MMC for a peritoneal cancer index (PCI) of 26. CRS included standardized peritonectomy and visceral resection related procedures. Regional intraperitoneal perfusion with HIPEC employed 40 mg MMC over 90 min, moderate hyperthermia (44 °C inflow, 42 °C outflow; closed abdomen technique).

**Figure 1:**
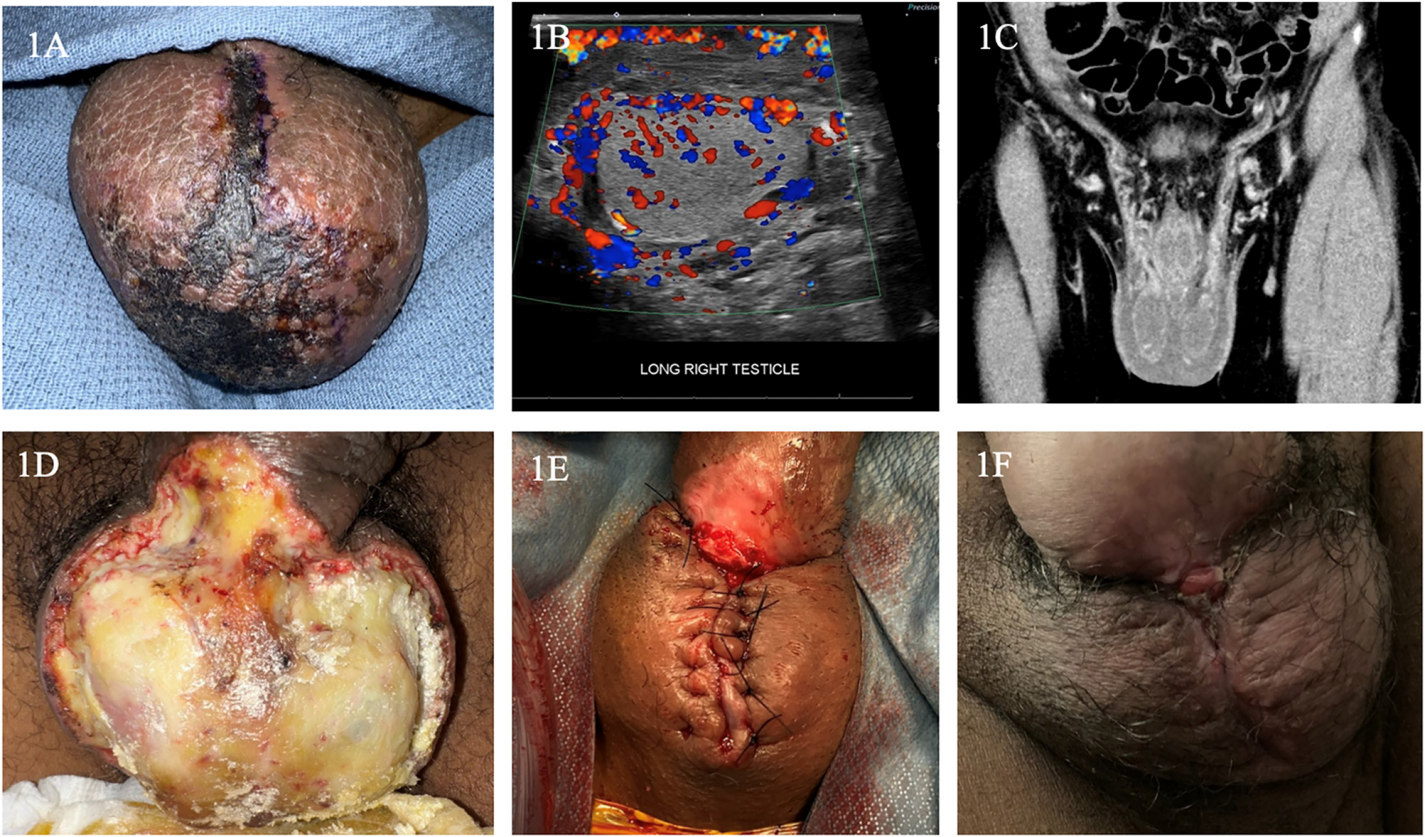
Pictorial timeline: Mitomycin C-induced full thickness genital cutaneous necrosis, diagnosis and treatment. (A) Genital cutaneous necrosis that initially developed along median raphe to fulminant bilateral scrotal involvement and eschar formation as viewed anteriorly on POD 75. (B) Ultrasound image on initial presentation with scrotal pain demonstrating thickened, hyperemic scrotal skin and lymphedema with normal testes. (C) Coronal image from CT with IV contrast demonstrates retroperitoneal infiltration along the testicular veins (red arrowheads), one hypothesized pathogenesis of genital necrosis. (D) Scrotal wound after multiple debridement’s of full thickness genital cutaneous necrosis on POD 81 following complete demarcation. Notable underlying tunica albuginea and testicles intact without evidence of further necrosis or infection. (E) Scrotal wound status post primary closure POD 185. (F) Scrotal wound as seen on POD 237, approximately 7 weeks following primary closure, demonstrating excellent healing and cosmesis.

Post-operative course was unremarkable. On postoperative day (POD) 60, patient described new onset neuropathic type scrotal pain, swelling and erythema. Cellulitis presumed and treatment with a short course of antibiotics ensued. Thereafter, he re-presented on POD 75 with worsening pain and progressive skin necrosis involving the penoscrotal junction tracking along the median raphe of the anterior scrotum (Figure 1A). Laboratory profile demonstrated a leukocytosis to 14.6 white blood cells/μL, an absolute neutrophil count of 8.05 and thrombocytosis to 479,000 platelets/μL. Scrotal ultrasound revealed thickened, hyperemic scrotal lymphedema and normal testes (Figure 1B). CT imaging suggested orchitis with retroperitoneal infiltration along the testicular veins (Figure 1C). He developed interval purulent cellulitis requiring scrotal debridement on POD 81 (Figure 1D). Postoperative wound care included negative pressure therapy subsequently deferred for local topical dressings. His wound continued to granulate over several months and thereafter underwent elective debridement and primary closure on POD 185 (Figure 1E). Excellent healing and cosmetic result noted POD 237 (Figure 1F).

Rarely reported, delayed genital cutaneous necrosis represents a serious, debilitating complication after CRS/HIPEC with regional perfusion using MMC. Estimated incidence approximates 1.5 % [1], [2], [3], [4]. At presentation, a broad differential diagnosis includes infectious etiologies such as Fournier’s gangrene, inflammatory diseases such as pyoderma gangrenosum, and vasculitis, all likely contributing to underreporting, thus masquerading the true sequela believed to represent inadvertent extravasation of MMC causing cutaneous genital necrosis.

The mechanism and pathophysiology leading to delayed scrotal necrosis remains unclear. MMC is a DNA-alkylating vesicant chemotherapeutic agent known to cause local skin and soft tissue injury. Currently theorized, patients with a patent processus vaginalis (PPV) may be at higher risk due to local passage of MMC through the inguinal canal into the scrotum, although the patient presented herein had no evidence of inguinal hernia, nor PPV, while necrosis appeared to course along the midline raphe predominantly involving the scrotal rugae.

MMC extravasation from surgically created anatomical connections is a plausible mechanism for injury in this patient with retroperitoneal infiltration and lymphedema. However, this theory does not explain the delayed nature in which scrotal skin necrosis occurs [1], [2], [3], [4]. Postulates include MMC’s ability to chronically bind cellular DNA with a “recall mechanism” incited by an event, such as genital dependent pressure, causing a symptomatic flare-up provoking hyperemic cutaneous venous irritation, lymphedema and resultant skin necrosis [5]. Another theory suggests thrombotic microangiopathy secondary to dose dependent toxicity of MMC. Thrombocytosis, as seen in our CRS, post splenectomy patient, reportedly occurs in >50 % of patients at the time of presentation with delayed genital necrosis [1]; however, absence of local vasculitis and thrombosis on pathologic review calls this genesis into question.

This presentation follows a small but consistent body of literature describing the development of delayed cutaneous genital necrosis following HIPEC with MMC. Extravasation of MMC by a PPV, although favored in the literature as an etiology for drug extravasation, deserves further investigation. Increased awareness and better understanding of this rare complication after CRS/HIPEC is tantamount to prevent and minimize associated morbidity in the future. Limitations include the retrospective nature of this communication, duration of follow-up and requirement for more patients to validate any postulations concerning disease etiology and treatment strategy.
